# *Lachemilla mexiquense* (Rosaceae), a new species from Mexico

**DOI:** 10.3897/phytokeys.62.7953

**Published:** 2016-03-25

**Authors:** Diego F. Morales-Briones

**Affiliations:** 1 Department of Biological Sciences and Stillinger Herbarium, University of Idaho, 875 Perimeter Drive MS 3051, Moscow, Idaho 83844-3051, U.S.A.

**Keywords:** *Lachemilla mexiquense*, *Lachemilla*, Rosaceae, Mexico, new species

## Abstract

A new species of *Lachemilla* (Rosaceae), *Lachemilla mexiquense* D.F. Morales-B., from Mexico is described and illustrated. This species is similar to *Lachemilla aphanoides* by its tripartite leaves and glomerulate inflorescence with entirely glabrous flowers, but it differs by its stonoliferous habit, persistent basal leaves and basal stipules, and smaller flowers with a campanulate-elongate hypanthium and single carpel. A key to the species of *Lachemilla* in Mexico is provided.

## Abstract (es)

Una nueva especie de *Lachemilla* (Rosaceae), *Lachemilla mexiquense* D.F. Morales-B., de México se describe e ilustra. Esta especie es similar a *Lachemilla aphanoides* por sus hojas tripartitas e inflorescencias glomeruladas con flores completamente glabras, pero difiere por su hábito estolonífero, hojas basales y estipulas basales persistentes y flores de menor tamaño con hipantio campanulado-elongado con un solo carpelo. Se provee una clave para las especies de *Lachemilla* en México.

## Introduction

*Lachemilla* Focke (Rydb.) is a morphologically highly variable group that includes perennial herbs, sub-shrubs, and shrubs. It comprises ca. 80 species and occurs between 2200 and 5000 m in elevation in the high mountains of the Neotropics, from northern Mexico to northern Argentina and Chile (Romoleroux 1996; Gaviria 1997), where it is one of the main elements of the páramo and superpáramo flora in South America. In Mexico the genus is represented by at least 10 species that can be found in sub-alpine and alpine habitats from the mountain pine forest to the high elevation zacotanales. *Lachemilla* has a nearly ubiquitous occurrence throughout the montane American tropics and remains a taxonomically complex group where species boundaries are often unclear and the infrageneric taxonomy is poorly defined. Since the only comprehensive revision of *Lachemilla* (Perry 1929), several works have tried to clarify its taxonomy (Rothmaler 1935, 1937; Notov and Kusnetzova 2004) and recently several regional treatments have been published (Romoleroux 2004; Barrie 2015), but a complete revision of the group is still needed. Recent taxonomic work aiming to produce a monographic treatment of *Lachemilla* has resulted in the description of several new species (Romoleroux 2009; Romoleroux and Morales-Briones 2012).

Here, a new species of *Lachemilla* is described and illustrated from Mexico. Material of the new species was collected in June 2015 during an expedition focusing solely on the genus *Lachemilla*. After detailed examination of the specimen, revision of species descriptions, and comparison with specimens at CAS, F, MEXU, MO, NY, TEX, and UC, it was established that the specimen collected in central Mexico represents a new species. The taxonomic treatment of this new species, including a key to species of *Lachemilla* in Mexico is provided.

## Taxonomic treatment

### Lachemilla mexiquense

Taxon classificationPlantaeRosalesRosaceae

D.F. Morales-B. sp. nov.

urn:lsid:ipni.org:names:77153916-1

Figures 1 , 2A

#### Diagnosis.

*Lachemilla mexiquense* differs from *Lachemilla aphanoides* (Mutis ex L. f.) Rothm. by its caespitose and stoloniferous habit, creeping stems, basal leaves and basal stipule persistent, campanulate-elongate hypanthium and the presence of a single carpel.

#### Type.

MEXICO. Estado de México, Municipio Ocuilan, 4 km NE of Santa Martha on road Santa Martha–Huitzilac, 19.07567°N, 99.36215°W, alt. 3,050 m, 30 June, 2015, *Morales-Briones D. F. & Tenorio-Lezama P. 683.* (holotype: ID!; isotype: MEXU!, QCA!).

#### Description.

Caespitose herbs, stoloniferous; stems creeping, mat-forming, branches sometimes rooting, pilose. Basal leaves 3-parted, 6–20 × 5–15 mm, chartaceous, lateral segments bifid, segments obovate to cuneate, margin incised-dentate, lower surface pilose, upper surface sparsely pilose to glabrescent; petioles 12–35 mm long; stipules 5–15 mm long, adnate to the petiole at base, free, entire and acute at apex, membranaceous, greenish-white. Stem leaves 3-parted, 7–12 × 4–7 mm, chartaceous, lateral lobes entire or bifid, segments obovate to cuneate, margin deeply cleft, lower surface pilose, upper surface sparsely pilose to glabrescent; petioles 3–5 mm long; stipules 3–8 mm long, adnate to the petiole at base, free at apex, 6-lobed at apex, membranaceous and greenish-white at base, chartaceous and green at apex. Inflorescences axillary or terminal, glomerulate, 6–10 flowered cymes; floral bracts lobed, spreading; pedicels 1–1.5 mm long, pilose at apex. Flowers 1.2–1.5 mm long; hypanthium campanulate-elongate 1–1.2 × 0.6–0.8 mm, glabrous outside, glabrous inside, green when young, reddish at maturity; episepals 4, ovate, 0.6–0.7 × 0.5–0.7 mm, glabrous, apex acute; sepals 4, lanceolate, 0.5–0.6 × 0.2–0.3 mm, glabrous, apex acute; stamens 2, filaments 0.2–0.3 mm long; carpels 1, stigma clavate. Achenes ovoid-globose, 0.9–1.1 × 0.6–0.8 mm, glabrous, one-seeded. Seeds ovate, 0.7–0.8 × 0.4–0.6 mm, pink, glabrous.

#### Distribution and ecology.

*Lachemilla mexiquense* is only known from the State of Mexico, municipality of Ocuilan, at ca. 3050 m altitude (Figures 2B, 3). *Lachemilla mexiquense* grows at the border of dense forest of various species of *Pinus*. This species lives in sympatry with *Lachemilla procumbens* (Rose) Rydb., *Lachemilla vulcanica* (Schltdl. & Cham.) Rydb., and *Lachemilla aphanoides* (Mutis ex L. f.) Rothm. It was collected in flower and fruit in late June.

**Figure 1.**
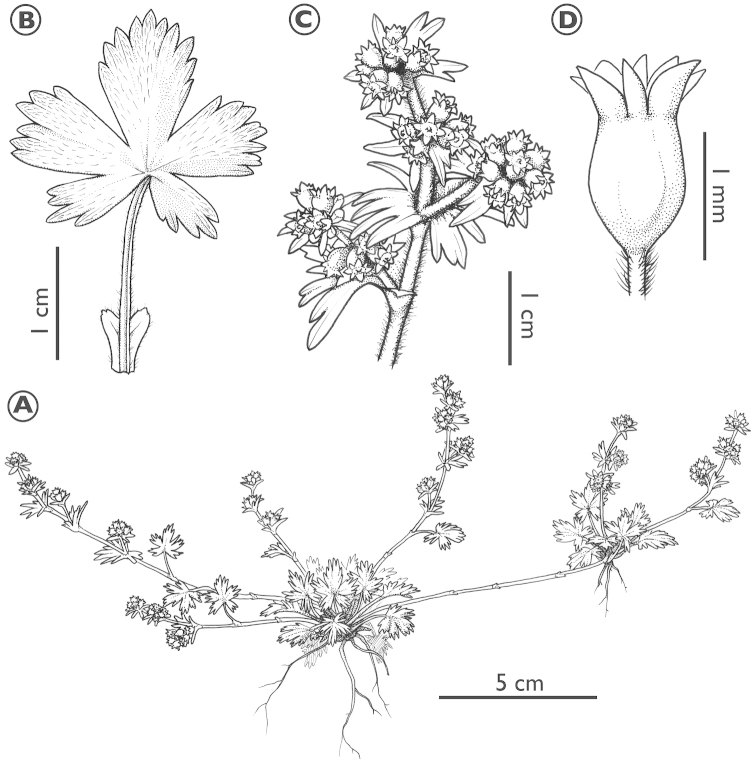
*Lachemilla mexiquense*. **A** Habit **B** Basal leaf and stipule **C** Flowering branch **D** Flower. Illustration by P. Lu-Irving.

**Figure 2.**
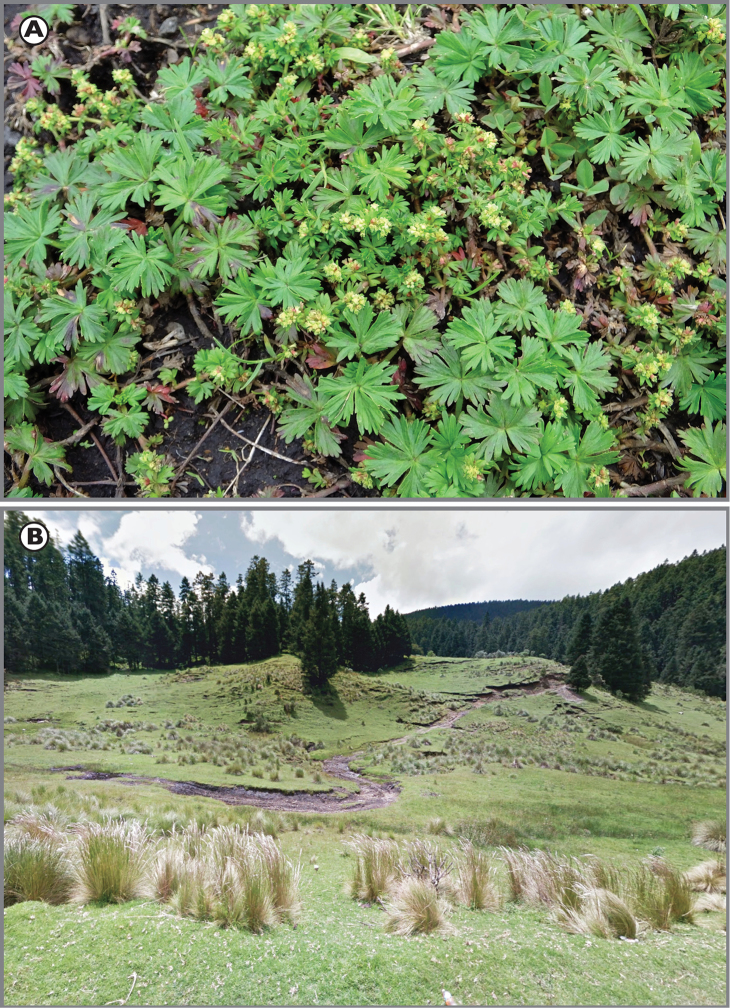
*Lachemilla mexiquense*. **A** Habit **B** Type locality.

**Figure 3.**
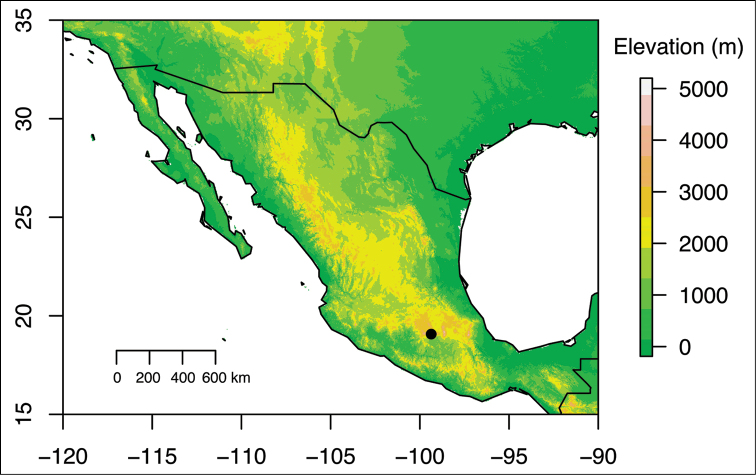
Geographic distribution of *Lachemilla mexiquense*.

#### Etymology.

The specific epithet refers to the demonym for State of Mexico where the type specimen was collected.

#### Conservation status.

*Lachemilla mexiquense* has a very limited geographic distribution, and is only known from the type locality (Figures 2B, 3). It occurs right outside the limits of the Cumbres del Ajusco National Park and Lagunas de Zempoala National Park. The type locality has been severely impacted by human activities, including conversion to agriculture (sheep and cow grazing). Following the IUCN (2014) guidelines, based on the reduced geographic distribution and altered land use at the type locality, this species should be categorized as endangered (EN), at least until other populations are discovered.

#### Notes.

*Lachemilla mexiquense* resembles *Lachemilla aphanoides* by having tripartite leaves with bifid lateral segments and glomerulate inflorescence with entirely glabrous flowers. Nevertheless, *Lachemilla mexiquense* differs from *Lachemilla aphanoides* by its caespitose habit, creeping stems, and stolons that form dense mats. Also, the basal leaves and basal stipules are persistent, and flowers are smaller (1.2–1.5 mm long) with a campanulate-elongate hypanthium and single carpel. *Lachemilla rupestris* (Kunth) Rothm., a species from Andean South America with similar habit, differs from *Lachemilla mexiquense* by having entire lateral segments of the leaves, yellow-brown membranaceuos basal stipules, and larger flowers (2.5–3 mm long) with a turbinate-campanulate hypanthium, sericeous-hirsute pubescence, and 2–4 carpels.

In addition, phylogenetic analyses of chloroplast and nuclear DNA (Morales-Briones et al. unpubl. data) clearly separate *Lachemilla mexiquense* from *Lachemilla aphanoides* and *Lachemilla rupestris*. The chloroplast phylogeny place it as sister species of the ‘Orbiculate group,’ which encompasses species with stoloniferous habit, palmately lobed or cleft leaves, and flowers disposed in profuse terminal cymes, like *Lachemilla pectinata* (Kunth) Rothm. The nuclear phylogeny fails to confidently resolve the phylogenetic position of *Lachemilla mexiquense*, suggesting that it may be of hybrid origin, a common pattern seen throughout *Lachemilla*.

### Key to the species of *Lachemilla* in Mexico

**Notes.** Adapted from Standley and Steyermark (1946), Romoleroux (2004), and Barrie (2015). Accepted taxa and synonymy follows the regional revisions of Romoleroux (2004) and Barrie (2015), with the exception of *Lachemilla sibbaldiifolia* (Kunth) Rydb. and *Lachemilla pringlei* Rydb., which based on extensive field observations and the examination of herbarium material, are considered here as two distinct taxa.

**Table:** 

1	Leaves pinnately divided	***Lachemilla pinnata***
–	Leaves simple or palmately divided or cleft	**2**
2	Basal leaves 5–11-lobed or 5–7-cleft	**3**
–	Basal leaves 3–5-cleft or 3–5-parted	**4**
3	Leaves shallowly 5–11-lobed, lobes triangular	***Lachemilla pectinata***
–	Leaves deeply 5–7-cleft, lobes elliptical to ovate	***Lachemilla venusta***
4	Inflorescence of loose cymes; hypanthium pubescent within	**5**
–	Inflorescence glomerulate, forming dense cymes; hypanthium glabrous within	**6**
5	Leaves 3-parted with bifid lateral segments, appearing 5-parted; stipules lobed or incised-dentate	***Lachemilla procumbens***
–	Leaves 3-parted with entire lateral segments, not appearing 5-parted; stipules bifid	***Lachemilla vulcanica***
6	Plants pilose to glabrate; flowers glabrous	**7**
–	Plants hirsute to sericeous; flowers pubescent, sometimes glabrate with age	**8**
7	Stems decumbent, ascending or erect; basal leaves and basal stipules often caduceus; flowers 1.5–3.0 mm; 1–3 carpels	***Lachemilla aphanoides***
–	Stems creeping, stoloniferous; basal leaves and basal stipules persistent; flowers 1.2–1.5 mm; 1 carpel	***Lachemilla mexiquense***
8	Hypanthium densely pubescent with very short hairs; lower leaves short-petioled, the upper leaves sessile	***Lachemilla velutina***
–	Hypanthium sparingly pubescent with appressed hairs, lower and upper leaves petioled	**9**
9	Leaves appearing 5-lobed, the lateral lobes bifid; achenes subacute or subobtuse	***Lachemilla sibbaldiifolia***
–	Leaves appearing 3-lobed, the lateral lobes not bifid; achenes tapering to an acute apex	***Lachemilla pringlei***

## Supplementary Material

XML Treatment for Lachemilla mexiquense
